# Clinical and Biochemical Correlation With Cytomorphological Findings of Lymphocytic Thyroiditis: An Experience at a Tertiary Centre in the Himalayan Foothills

**DOI:** 10.7759/cureus.24127

**Published:** 2022-04-13

**Authors:** Monika Singh, Suman Kumari, Bhoomika Kaushik, Arvind Kumar, Ashok Singh, Ravi Hari Phulware, Pakesh Baishya, Prashant Durgapal, Nilotpal Chowdhury, Shalinee Rao, Sanjeev Kishore, Bela Goyal

**Affiliations:** 1 Department of Pathology and Laboratory Medicine, All India Institute of Medical Sciences, Rishikesh, IND; 2 Department of Biochemistry, All India Institute of Medical Sciences, Rishikesh, IND

**Keywords:** thyroid cytology, aiims rishikesh, uttarakhand, prevalence, epidemiology and public health, biochemical biomarker, fine needle aspiration cytology (fnac), himalayan region, lymphocytic thyroiditis, cytomorphology

## Abstract

Introduction

Lymphocytic thyroiditis is an autoimmune disorder and one of the major causes of hypothyroidism. On cytomorphology, it is characterized by follicular destruction by lymphocytes with elevated biochemical markers, including a panel of autoantibodies against thyroid antigens. This study aimed to determine the prevalence of various cytological features of lymphocytic thyroiditis and their correlation with clinical presentation and biochemical parameters.

Materials and methods

We conducted a hospital-based cross-sectional study of 105 patients diagnosed with lymphocytic thyroiditis on cytology at our tertiary care center in the Himalayan foothills from December 2018 to December 2019. We recorded and analyzed baseline demographic characteristics, clinical features, and biochemical parameters to assess correlations between cytological findings and grades.

Results

The study included 105 patients with lymphocytic thyroiditis (90 females, 15 males). The study population age ranged from 11 years to 80 years, with the disease most common in patients aged 21 to 40 years. Grade II was the most common cytological presentation (n=65, 62%). Thyroid-stimulating hormone levels were elevated in 33.3% of cases, and anti-thyroid peroxidase levels were elevated in all 25 cases for whom data were available (p>0.05).

Conclusion

Cytological diagnosis of lymphocytic thyroiditis was compatible in all cases in the study. However, cytological grading did not correlate with the clinical presentation and biochemical parameters. The diagnosis of lymphocytic thyroiditis could be missed if clinicians use clinical findings and biochemical parameters alone.

## Introduction

Thyroid disorders are among the most prevalent endocrine diseases worldwide. Even in low-income countries like India, approximately 3.2% of the population is affected by thyroid-related problems [1,2]. Thyroid disorders are promptly diagnosed due to the thyroid gland's size and easy accessibility for clinical examination. A wide range of investigations can diagnose thyroid disorders, such as various hormone assays, antibodies, fine needle aspiration cytology (FNAC), and ultrasonography (USG) [3].

Hypothyroidism is the most prevalent thyroid disorder in the post-iodized era [3]. Iodine supplementation has increased the prevalence of lymphocytic infiltration of thyroid tissue and thyroid antibody production for unknown reasons [4]. Due to a lack of pre-iodine supplementation prevalence data, conclusive remarks on this subject are impossible. In India, lymphocytic thyroiditis is the most common cause of hypothyroidism in sufficient iodine uptake states [5-7]. The prevalence of lymphocytic thyroiditis ranged from 7.6% to 15.3%, according to various hospital-based Indian studies [8-10]. Most authors use the term lymphocytic thyroiditis synonymously for autoimmune thyroiditis, including both goitrous and non-goitrous thyroiditis [11]. Like other autoimmune diseases, it is also more commonly seen in women and can present clinically as diffuse or nodular goiter or an atrophic non-goitrous form depending on its clinical phase. FNAC is the investigation of choice for diagnosing lymphocytic thyroiditis and is further supported by serological studies of antibodies, thyroid hormone profile, radioactive iodine uptake, and USG [12].

As a primary investigation for thyroid swelling, FNAC offers detailed morphological study, grading of thyroiditis based on the extent of infiltration, and correlation of morphology with various biochemical parameters and radiological findings. This study aims to comprehensively describe the prevalence of lymphocytic thyroiditis with clinical presentation, serological findings, and cytological correlations in North India and the Himalayan region. Given a thorough search of the literature, the present study is the only such study to do so.

## Materials and methods

We conducted a retrospective observational study of 105 patients who visited the department of pathology at a tertiary care institute from December 2018 to December 2019 and were diagnosed with lymphocytic thyroiditis on FNAC smears. We excluded USG-guided FNAC cases and patients with a history of exogenous thyroid intake or thyroid surgery. The cytomorphological criteria for diagnosis were entanglement or infiltration of lymphocytes/plasma cells to follicular epithelial cells/follicles, a significant increase in lymphocyte/plasma cell levels, Hurthle cell change, mild to moderate anisonucleosis, hemorrhagic background with or without thick colloid. Two pathologists graded thyroiditis by evaluating the smears prepared using Bhatia et al.'s criteria [12]. For FNAC, we used 23-gauge needles and 10-mL syringes. We used aspiration and non-aspiration techniques with at least two pricks from different sites. The smears are fixed in ethanol and stained with May Grunwald Giemsa and Papanicolaou stain. Thyroid profile parameters (T3, T4, and thyroid-stimulating hormone [TSH]) and anti-thyroid peroxidase (anti-TPO) antibody analysis were done using serum samples by chemiluminescent immunoassay.

We recorded clinical presentation, thyroid profile (T3, T4, and TSH), morphological features, and radiological findings of the lesions. We correlated the cytological grades with various parameters and assessed for statistically significant correlations. Anti-TPO antibody levels were not available for many patients, so it was not included in the correlation study. We used the Mann-Whitney-Wilcoxon Test and the Chi-squared test to evaluate statistical correlation, with significance indicated by p<0.05 by using software SPSS statistics 28.0 (IBM Corp., Armonk, NY, USA). This study was approved by the ethical committee of All India Institute of Medical Sciences (AIIMS/IEC/22/99).

## Results

The study included 105 patients with lymphocytic thyroiditis Ninety were females (85.7%), and 15 were males (14.2%). Patients in the study ranged in age from 11 years to 80 years, and the disease was most prevalent in those aged 21 to 40 years (Table 1).

**Table 1:** Prevalence of various cytomorphological features studied

Features	Number of cases, n (%)
Adequate cellularity	95 (90%)
Follicular lymphocytic infiltration	94 (90%)
Hurthle cells	53 (50%)
Anisonucleosis	29 (27.6%)
Macrophages	14 (13%)
Plasma cells	7 (6.7%)
Giant cells	15 (14%)
Colloid absent	52 (49.5%)

Almost all patients presented with goiter; we noted 57 cases (54.2%) of diffuse goiter, 18 cases (17%) of multinodular goiter, and 31 cases (29.5%) of solitary nodules. Hormonal assay data were available for 75 patients. Of those, 37 cases (49.3%) were euthyroid, 26 cases (34.7%) were hypothyroid, and 12 cases (16%) had hyperthyroidism. Anti-TPO levels were available in only 25 cases, and all were positive. The limited availability of anti-TPO values precluded correlation with other parameters. The presence of Hurthle cells was the most prevalent cytological feature except for lymphocytic infiltration of follicles, as shown in Table 1.

The distribution of various cytological grades is shown in Table 2, and Grade II lymphocytic thyroiditis was the most common pattern observed in both females and males. Table 3 presents a comparison of TSH levels and cytological grades. The cytological grades were not significantly correlated with clinical and biochemical parameters (p>0.05). 

**Table 2:** Distribution of cases according to age, sex, and cytological grade

	Number of cases(n)					
Age	Grade I		Grade II		Grade III	
	Male	Female	Male	Female	Male	Female
≤20 years	1	3	2	6	-	-
21-40 years	2	17	4	27	-	4
41-60 years	2	8	2	20	-	-
≥ 60 years	1	1	1	3	-	1
Total	35		65		5	

**Table 3:** Comparison of thyroid-stimulating hormone (TSH) values with various cytological grades

	Grade I, n	Grade II, n	Grade III, n
TSH Higher than Reference Range	4	20	1
TSH Within Reference Range	14	18	1
TSH Below Reference Range	6	10	1

## Discussion

According to Bhatia et al., the first case of chronic thyroiditis was reported by Hakaru Hashimoto in 1912 as struma lymphomatosa [12]. Lymphocytic thyroiditis is synonymous with autoimmune thyroiditis and Hashimoto's thyroiditis which bears the name of its first reporting author [13]. Lymphocytic thyroiditis is an autoimmune disorder characterized by the activation of CD4+ T cells, which recruit many auto-reactive B cells which secrete various autoantibodies. Antibodies against thyroid peroxidase and thyroglobulin are the most specific biochemical markers for lymphocytic thyroiditis [13-15]. Many authors have stated the role of continued iodine supplementation in inducing lymphocytic thyroiditis in the post-iodized era [4,16-18]. Marwah et al. studied the prevalence of goiters in India's different states and union territories even after adequate iodine prophylaxis and found that goiters had a 30% prevalence and no significant decrease. The presence of various additional factors like urinary thiocyanate concentration and thyroid autoimmunity was likely part of goitrogenesis [19].

Like other autoimmune diseases, a genetic predisposition has been suggested in the development of autoimmune thyroiditis, especially in the hilly areas with lower iodine soil contents and more consumption of goitrogens [20,21]. Kumar et al. studied 105 patients with thyroid disorder from the Himalayan range of the Pauri Garhwal region over five years. The found lymphocytic thyroiditis in this hilly region was the second most common (27.27%) thyroid disorder after goiters (45%) [22]. There are no nationwide programs to monitor the prevalence of autoimmune thyroiditis in hilly areas. According to Nguyen et al. and Vanderpump et al., lymphocytic thyroiditis can affect patients of any age group, but most people are affected in the third and fourth decades of life, which aligns with our results [5,23]. However, this finding contrasted with a few western studies in which more prevalence was noted in the older groups [24]. No concrete study has been done on this association in India as of this writing. In our study, lymphocytic thyroiditis presented earlier and with increased severity in females, as seen in Table 1, which is in accordance with a previous study [25].

Lymphocytic thyroiditis can present clinically as a nodular goiter in its early stages or as diffuse swelling or atrophic form in the later stages. We found diffuse goiter among 48% of our population, while nodular goiter was seen in almost 46% of the patients, contrasting with other Indian studies where most cases had diffuse goiter (70% to 80% of cases) [8,13,23]. However, a nodular presentation was more prevalent in a few western studies than in our study [25]. Nearly half of the patients (49.3%) who presented in the early stages of the disease were euthyroid, whereas 34.7% of cases were suffering from hypothyroidism, and 16% had hyperthyroidism. Other studies report nodular goiter as the early clinical presentation, as in our study; Anila et al. and Sharma et al. reported a predominance of cases presenting in the earlier stages of the disease [7,25].

Cytological findings in the diagnosis of lymphocytic thyroiditis cases exhibited mostly adequate (95%) cellularity with an absence of colloid in 46% of cases. Follicular infiltration by lymphocytes was observed in 90% of cases, with features like anisonucleosis in 28%, Hurthle cells in 56%, and giant cells in 14% of cases. Plasma cells were seen in only 6% of cases. Cytological grading was done according to Bhatia et al. [13], and 32% of cases showed features of Grade I thyroiditis (i.e., few lymphoid cells infiltrating the follicles or increased number of lymphocytes in the background). Features consistent with Grade II thyroiditis exhibited mild to moderate lymphocytic infiltrate and additional features such as Hurthle cell, giant cells, and anisonucleosis in 62% of cases. At the same time, features of Grade III thyroiditis were noted in only 6% of cases in the form of florid lymphocytic infiltration with occasional follicular epithelial cells. Most previous authors reported a similar distribution pattern of various cytological grades in the patients, with features of Grade II being the most common [23,25].

In contrast to our results, Anila et al. observed that Grade I was the most common presentation in their study [7]. While the cytological findings are characteristic for diagnosing lymphocytic thyroiditis, our study found no significant correlation with clinical and biochemical parameters. Similar findings were also noted in some of the most prominent studies performed in the Himalayan region (Table 4).

**Table 4:** Comparison of present study with previous studies in the Himalayan region

Author	Study Duration	Number of Cases	Age (Years)	Sex (Male/Female)	Clinical presentation	Hormonal changes	Grading (%)		
							I	II	III
Sharma et al. [25]	1 year	52	10-80	6/46	-	65.4%	15.4	80.8	3.8
Iha et al. [15]	-	31	13-66	1/30	Diffuse: 87%; Nodular: 13%	-	9.7	67.7	5
Present study	1 year	105	12-70	15/95	Diffuse: 48%; Nodular: 46.7%	65%	33.3	62	4.7

While FNAC may be the gold standard method for diagnosing lymphocytic thyroiditis, it is limited by factors such as dilution by blood, number of aspirates, and individual microscopic observation. FNAC aspirates are obtained from a tiny parenchymal area and may not give the complete picture of changes present in the other regions.

## Conclusions

In all cases, the lymphocytic infiltration of thyroid follicles was pathognomonic, but when the cytological grades were compared with clinical and hormone assay values, we found no significant correlation. Therefore, the diagnosis of lymphocytic thyroiditis can be missed if clinical findings and biochemical parameters are used alone. Cytological findings and clinical, serological, and radiology should be used to reach a final, conclusive diagnosis. A more extensive population-based study is needed that uses the National Iodine Deficiency Disorders Control program and FNAC; such a study would be vital in diagnosing lymphocytic thyroiditis in resource-limited laboratories located in remote hilly regions. 
